# Provision of supportive care by an NGO in the face of a dual challenge: cancer and wartime

**DOI:** 10.1007/s00520-024-09009-w

**Published:** 2024-11-18

**Authors:** L Hamama, S Kuperman, M Bar-Doron, Y Hamama-Raz

**Affiliations:** 1 The Bob Shapell School of Social Work, Tel Aviv University, Ramat Aviv, Tel Aviv, Israel; 2 Halasartan (Stop Cancer) NGO, Tel Aviv, Israel; 3 School of Social Work, Ariel University, Ariel, Israel

**Keywords:** Nongovernmental organization, War, Cancer patients/survivors, Self-efficacy, Social support

## Abstract

**Purpose:**

In this study, we explored the work of Halasartan (Stop Cancer), an Israeli nongovernmental organization (NGO) and unique social support network for cancer patients and survivors aged 18–44, during a war period. Drawing on the conservation of resources (COR) theory, we examined whether self-efficacy, social support, psychological distress, and participation in activities that were geared toward alleviating the war situation at Time 1 (T1) would predict engagement in such activities at Time 2 (T2).

**Methods:**

A longitudinal design with two time-points was used, and NGO members completed self-report questionnaires. At T1, the cohort comprised 250 members (cancer patients/ survivors); at T2, there were 213. However, only 90 NGO members completed the questionnaires at both time-points.

**Results:**

A significant reduction in psychological distress was observed over time among participants engaged in the NGO’s activities, but no differences were observed in participants’ self-efficacy or social support. Moreover, participation in NGO activities during wartime at T2 was predicted by biological sex (female), lower self-efficacy, and participation in NGO activities at T1.

**Conclusion:**

Halasartan (Stop Cancer) played a pivotal role in offering a sense of normalcy, community, and support to young-adult cancer patients and survivors during a period of war. The study underscores the essential nature of NGO activities tailored to the unique needs of this demographic, particularly in times of crisis. A broader implementation of such supportive interventions to enhance the well-being of vulnerable populations is suggested.

## Introduction

Cancer patients and survivors face many challenges, ranging from the long-term consequences of treatment to the psychological and social implications of living with and beyond cancer [1, 2]. As such, it is reasonable to assume that a war might introduce additional stressors. Indeed, Ahmed [3] contended that in periods of conflict, both healthcare professionals and patients face mental strain, which can significantly impact the standard of care provided. Moreover, cancer patients/survivors may experience wartime disruptions in physical health, emotional well-being, social relationships, and overall functioning [4, 5].

Given that access to cancer treatment and supportive care services might be challenging during wartime (e.g., transportation difficulties, fears of leaving home, disruptions to healthcare infrastructure), nongovernmental organizations (NGOs) could play an important role. Feuerstein and Ganz [6] argued that quality of care throughout the cancer journey could indeed be optimized through complementary community support such as non-profit community-based organizations: support that could become even more critical in a time of war. Several researchers have noted that perceived organizational support is crucial for alleviating the negative impacts of stress or trauma. Such support systems enhance resilience, mental health, and well-being, particularly during traumatic events or widespread crises [7, 8].

In the current study we examined, over time, the Israeli NGO Halasartan (Stop Cancer), a social support network for cancer patients and survivors aged 18–44. Halasartan provides a comprehensive support system, addressing issues such as loneliness, maintaining relationships, fertility, career challenges, financial independence, and young parenthood (https://www.stop-cancer.co.il/a-spark-of-life-for-the-young/). This NGO offers movement therapy (both in-person and remotely via Zoom), yoga, therapeutic climbing, workshops on topics such as sexuality, video therapy, and creative writing, and emotional support groups for its community members—both cancer patients and survivors. However, following the October 7th, 2023 massacre in Israel, and the ensuing Swords of Iron War, these activities were swiftly modified to meet the new circumstances (i.e., from October 10 to December 28, 2023).

Drawing on the Conservation of Resources (COR) theory as outlined by Hobfoll, [9, 10] which highlights the importance of personal, social, and material resources that individuals endeavor to obtain, retain, and protect, the aim of this study was to explore factors that might predict engagement in activities developed during a war, over time. We thus focused on self-efficacy (a personal resource) and social support (an environmental resource) as predictors of such engagement.

Self-efficacy, the belief in one’s ability to organize and carry out the actions necessary to manage future situations [11], is pivotal in determining how individuals perceive and react to stress [12]. Individuals with higher self-efficacy, which enhances emotion regulation and encourages the seeking of social support, tend to view challenges as opportunities rather than threats [13, 14]. Moreover, high self-efficacy promotes resilience and a proactive approach to facing challenges. Low self-efficacy, by contrast, can result in avoidance, feelings of helplessness, and withdrawal [13]. It should be noted that the importance of social support and emotional well-being in developing a strong sense of self-efficacy is highlighted in the theory of self-efficacy [11].

Social support, the provision of psychological and material resources by social networks to help an individual manage stress [15], is divided into three main types: instrumental (e.g., tangible aid such as financial help or assistance with daily tasks), informational (e.g., offering advice or guidance to overcome challenges), and emotional (e.g., empathy, caring, reassurance, and trust) [15]. Social support, drawn from family, friends, neighbors, and institutions [16], is essential for improving psychological dynamics and overall well-being and serves as a protective buffer against various stressors [15, 17]. Studies consistently indicate a positive link between social support and better mental and physical health outcomes [16, 18, 19]. Additionally, in line with COR theory [9], social support can expand an individual’s resource pool and replenish or bolster other deficient resources.

As stated, in this longitudinal study, we highlight the case of an NGO that modified its activities to deliver essential services amidst the dual challenges of cancer and war. The focus of previous studies has primarily been on the impact of war on oncology in the Middle East and other conflict-affected regions [20, 21], regarding the obstacles to cancer care in these circumstances [22, 23]. In this study, however, we draw attention specifically to the voluntary sector’s role in supporting cancer patients/survivors during conflict. Thus, based on COR theory [9], the study’s purpose was twofold: (1) exploring differences in psychological distress, self-efficacy, social support, and engagement with those Halasartan activities that were geared toward alleviating wartime stress, over two time-points, among cancer patients/survivors, and (2) examining predictors (i.e., gender, psychological distress, self-efficacy, social support, and participation in the previously alluded-to wartime activities at T1) that might explain engagement in activities at T2.

## Methods

### Participants

The T1 cohort included 250 members of the Halasartan community (cancer patients/survivors). Their mean age was 36.22 (SD = 8.73); women constituted the majority (80%, *n* = 200); and over half were married or in relationships (66%, *n* = 164). Most identified as secular (72%), and 45% were unemployed. The mean years of education was 15 (SD = 3.0).

Regarding their medical situation, 36% were undergoing active therapy at the time of the study; 40% (*n* = 101) were receiving psychological treatment; and approximately 33% reported using antidepressants due to their cancer. Regarding NGO activities specifically related to the war situation, 18% participated in movement therapy via Zoom; 14% attended emotional support meetings via Zoom; 14% listened to podcasts; 4% practiced mindfulness; and 6% practiced yoga.

The T2 cohort (6 weeks after T1) included 213 members of the Halasartan community. Their mean age was 35.16 (SD = 6.87); most were women (71%, *n* = 151); and more than half were married or in relationships (62%, *n* = 132). Most described themselves as secular (77%), and 41% were unemployed. The mean years of education was 15 (SD = 3.0).

As for their medical condition, 34% were in active therapy at the time of the study; 44% (*n* = 94) were receiving psychological treatment; and about 44% reported the use of antidepressants due to their cancer. Regarding NGO activities specifically related to the war situation, 17% participated in movement therapy via Zoom; 26% participated in emotional support meetings via Zoom; 15% listened to podcasts; 13% practiced mindfulness; and 12% practiced yoga.

After combining both cohorts, the sample consisted of 90 participants who completed the questionnaires at both time-points. Their ages ranged from 21 to 41 (M = 35, SD = 6.9); most were women (77%, *n* = 69); more than half were married or in relationships (59%, *n* = 53); most identified as secular (66%); and 48% were unemployed. The mean years of education was 15 (SD = 3.8). As for their medical condition, 26% were in active therapy at the time of the study; 48% (*n* = 43) were receiving psychological treatment; and about 30% reported the use of antidepressants due to their cancer. Regarding NGO activities specifically related to war situation, 30% participated in movement therapy via Zoom; 29% participated in emotional support meetings via Zoom; 18% listened to podcasts; 14% practiced mindfulness; and 16% practiced yoga.

### Procedure

The study received approval from the institutional review board of the first author’s university (research no. 0007450–1) and was conducted at two time-points: T1, from October 25 to November 5, 2023 (during this period, there were rocket barrages on Israel that made it impossible to carry out routine activities), and T2, from December 5 to 22, 2023 (after a reduction in the firing of missiles). Participants were recruited through Halasartan. Specifically, a direct link to an electronic questionnaire hosted on Google Forms was shared via a posted Hebrew message on the NGO’s Facebook page and further disseminated through their WhatsApp contacts. Eligibility criteria included membership in Halasartan, being aged 18–44, and fluency in reading Hebrew. Participants were informed about the purpose of the study and provided their consent electronically by selecting “I agree to participate.”

### Measures

Participants completed the following self-report questionnaires:

- *Personal data*: Biological sex, age, marital status, number of children, employment status, and degree of religiosity. Participants were also asked to indicate whether they were in active medical treatment (yes/no), what the current medical treatment was (i.e., chemotherapy, radiotherapy, hormonal therapy, biological therapy), and the duration of being a cancer survivor. Additionally, they were asked to indicate whether they were receiving psychological treatment (yes/no) and whether they were currently using an antidepressant or anti-anxiety medication due to their cancer.
- *Self-efficacy*: Assessed via the Hebrew-language version of the New General Self-Efficacy questionnaire (NGSE [24]), a self-report instrument consisting of eight items such as: “I will be able to successfully overcome many challenges.” Each item is rated on a 5-point Likert scale ranging from 1 (*strongly disagree*) to 5 (*strongly agree*). Responses are summed for total scores, ranging from 8 to 40. Higher scores indicate greater perceived self-efficacy. The authors of the original questionnaire were the ones who also compiled the Hebrew-language version of the NGSE[24], which is known for its robust psychometric properties, including a test–retest reliability of *r* = 0.86 and an internal reliability of *α* = 0.86. Consequently, the Hebrew-language version was utilized in the current study. The internal reliability in this study was *α* = 0.96 at T1 and *α* = 0.96 at T2.
- *Social support*: Assessed through the Multidimensional Scale of Perceived Social Support (MSPSS [25]), which consists of three subscales distinguishing between social support sources: family, friends, and significant others. The MSPSS includes 12 items (four for each subscale; e.g., “My family really tries to help me”) based on a 7-point Likert scale from 1 (*very much disagree*) to 7 (*very much agree*). The total score is the sum of all items, ranging from 12 to 84. For the original questionnaire [25], internal reliability was *α* = 0.88 for the first measurement and *α* = 0.85 for the second measurement. In the present study, Cronbach’s alpha was 0.95 for T1 and 0.95 for T2.
- *Psychological distress*: Measured by the Kessler Psychological Distress Scale (K6 [26]), which consists of six items that examine manifestations of nonspecific psychological distress: nervousness, hopelessness, irritability, negative affect, fatigue, and worthlessness, experienced over the past 30 days (e.g., “During the last 30 days, about how often did you feel nervous?”). Participants were asked to use a 5-point scale from 0 (*none of the time*) to 4 (*all the time*), with total scores ranging from 0 to 24, and higher scores indicating higher levels of psychological distress. Cronbach’s alpha for the original scale [27] ranged from 0.89 to 0.92. In the present study, Cronbach’s alpha was 0.86 for T1 and 0.87 for T2.

*NGO activities aimed to alleviate wartime stress*: Participants were asked to indicate whether they utilized the following activities: movement therapy group, emotional support group, a podcast recorded especially for the period, mindfulness, and yoga (yes/no). All activities were available through Zoom to Halasartan community members.

### Statistical analysis

Statistical analyses were conducted using R software [28] (Version 4.2.3) in conjunction with R-Studio user interface [29] (Version 2023.6.1.524). Specifically, mean comparison tests between the time measurements were calculated by employing multiple *t*-tests for dependent samples. Relations between variables at the same time were examined using Pearson’s pairwise correlations, and the effects of T1 on T2 participation were analyzed through ordinary least squares regression (OLS). The hierarchical OLS regression model consisted of four steps: biological sex was included as a predictor in the first step to control for a possible effect; self-efficacy and social support were added as independent variables (IVs) in the second step; psychological distress was added as an IV in the third step; and, in the fourth step, participation at T1 was added as the last predictor.

## Results

### Differences in the main study measures between the two time-points

Table 1 presents the means, standard deviations, and statistics regarding the differences between *T1* and* T2*. According to this table, significant differences between T1 and T2 were observed only in psychological distress. Specifically, psychological distress at T2 was significantly lower than at T1. No other significant differences were found at T2.

**Table 1:** Differences in main study measures between the two time-points

Variable	Time	*M*	*SD*	*t(89)*	*p*	*Cohen’s d*
**Psychological distress**	T1	2.82	0.89	6.68	< .001	0.47
	T2	2.41	0.87			
**Social support**	T1	3.7	0.99	0.28	0.78	0.03
	T2	3.68	1.05			
**Self-efficacy**	T1	3.84	1.3	− 1.38	0.17	0.12
	T2	3.99	1.13			

Note: ^T1^*N* = 250; ^T2^*N* = 213

### The intercorrelations between the main study measures at the two time-points.

The intercorrelations between the variables and activity participation at T1 are presented in Table 2. From this table, it can be observed that a weak positive correlation was found between psychological distress and participation (*r* =  − 0.24, *p* < 0.05), indicating that individuals engaging in activities aimed at alleviating wartime stress tended to experience higher levels of psychological distress. Additionally, social support and self-efficacy were negatively correlated with psychological distress, and social support was positively correlated with self-efficacy.

**Table 2:** Descriptive statistics and intercorrelations between the research variables at T1

Variable	*M*	*SD*	1	2	3
1. Activities	0.71	1.0			
2. Psychological distress	2.82	0.89	0.24*		
3. Social support	3.7	0.99	− 0.12	− 0.40**	
4. Self-efficacy	3.84	1.3	− 0.15	− 0.36**	0.48**

^*^p < .05, **p < .01

Note: *N* = 250

As for T2, Table 3 presents the intercorrelations between the variables and activity participation. According to this table, only self-efficacy was negatively correlated with participation (*r* =  − 0.26, *p* < 0.05), indicating that individuals engaging in activities meant to relieve wartime stress at T2 tended to experience lower levels of self-efficacy. Further, at T2 as well, social support and self-efficacy were negatively correlated with psychological distress, and social support was positively correlated with self-efficacy.

**Table 3:** Descriptive statistics and intercorrelations between the research variables at T2

Variable	*M*	*SD*	1	2	3
1. Activities	1.07	1.2			
2. Psychological distress	2.41	0.87	0.12		
3. Social support	3.68	1.05	− 0.06	− 0.51**	
4. Self-efficacy	3.99	1.13	− 0.26*	− 0.40**	0.39**

^*^p < .05, **p < .01

Note: *N* = 213

### Explaining participants’ engagement in activities meant to alleviate wartime stress at T2

To determine whether participants’ sex, self-efficacy, social support, psychological distress, and participation at T1 would predict activity participation at T2, an ordinary least squares (OLS) regression was calculated, as shown in Table 4. The observations in this table describe the final model’s coefficients, indicating the following: Women were more likely than men to participate (*β* = 0.34, *p* = 0.047); higher self-efficacy was associated with lower participation (*β* =  − 0.28, *p* = 0.001); and participation at T1 was positively related to participation at T2 (*β* = 0.68, *p* < 0.001).

**Table 4:** The model regression to predict T2 wartime activities (*N* = 90)

Step	Predictors	*b*	*SE*	*β*	*p*	*95% CI*	*R2*	*ΔR2*
Step 1	(Intercept)	0.62	0.26	− 0.37	**0.018**	0.11–1.13	0.043	0.032*
	Sex (female)	0.58	0.29	0.49	**0.05**	0.00–1.17		
Step 2	(Intercept)	1.82	0.54	− 0.31	**0.001**	0.74–2.91	0.146	0.116**
	Sex (female)	0.49	0.28	0.41	0.089	− 0.08–1.05		
	Self-efficacy	− 0.3	0.11	− 0.32	**0.006**	− 0.51– − 0.09		
	Social support	0.01	0.14	0	0.966	− 0.27–0.28		
Step 3	(Intercept)	1.93	0.84	− 0.31	**0.024**	0.26–3.60	0.146	0.106**
	Sex (female)	0.49	0.29	0.41	0.09	− 0.08–1.06		
	Self-efficacy	− 0.3	0.11	− 0.33	**0.006**	− 0.52– − 0.09		
	Social support	0	0.15	0	0.993	− 0.29–0.29		
	Psychological distress	− 0.03	0.15	− 0.02	0.866	− 0.33–0.27		
Step 4	(Intercept)	1.77	0.6	− 0.26	**0.004**	0.58–2.95	0.576	0.551***
	Sex (female)	0.41	0.2	0.34	**0.047**	0.01–0.81		
	Self-efficacy	− 0.26	0.08	− 0.28	**0.001**	− 0.41– − 0.10		
	Social support	0	0.1	0	0.991	− 0.21–0.20		
	Psychological distress	− 0.21	0.11	− 0.16	0.055	− 0.43–0.00		
	Activities at T1	0.81	0.09	0.68	** < 0.001**	0.64–0.99		

^*^p < .05, **p < .01, ***p < .001

Note: *N* = 90

The bold entries emphasize statistically significant findings

## Discussion

The focus of this study was on the Israeli NGO Halasartan, which routinely provides supportive care services for cancer patients and survivors. However, due to the war taking place in Israel, this NGO modified its activities to meet community members’ needs. Based on COR theory [9], we explored differences in psychological distress, self-efficacy, social support, and participation in activities meant to alleviate wartime stress at two time-points. Additionally, we examined predictors (i.e., gender, psychological distress, self-efficacy, social support, and participation at T1) that might explain participation at T2.

The study’s findings revealed two main points. First, differences were observed only in psychological distress, where psychological distress at T2 was significantly lower than at T1. This result confirms findings from a previous study in which it was shown that citizens of Sarajevo who stayed in Sarajevo during the war in Bosnia and Herzegovina reported a decrease in psychological symptoms and general psychological distress over time [30]. A possible explanation for this finding can be found in the term “habituation,” which refers to the process by which individuals become accustomed to stressors over time, and a reduction in the physiological response elicited by exposure to a repeated stressor takes place [31, 32]. Thus, it might be that cancer patients/survivors during the war in Israel habituated to the war-related stressors, especially given that Halasartan-designed activities focused on reducing distress (e.g., mindfulness, yoga, movement therapy group, and emotional support groups). Nevertheless, as only 90 members of the Halasartan community completed the questionnaires at both time-points, caution should be taken when interpreting this result.

The finding that there were no differences across time in participants’ self-efficacy or social support can be interpreted through COR theory [9]. Self-efficacy (a personal resource) plays a crucial role in how individuals navigate challenges and maintain well-being, and social support (a social/environmental resource) provides individuals with a support system that can help them cope with stress and adversity. That said, individuals who have resources are more capable of gaining, and gain begets further gain. By contrast, those who lack resources are more vulnerable to resource loss, and the initial loss breeds even more loss [33]. As such, it might be that during their cancer experience, participants gained resources, and these gains led to positive cycles of resource accumulation that limited the impact of wartime distress across time. Further, our study demonstrated that self-efficacy and social support were negatively correlated with psychological distress, and social support was positively correlated with self-efficacy, at both time-points. These findings align with previous studies among cancer patients/survivors [34–36], revealing a positive correlation between self-efficacy and social support.

Regarding participation in NGO activities meant to alleviate wartime stress, our findings indicated that members of the Halasartan community with higher levels of psychological distress participated at T1, whereas at T2 only those with lower levels of self-efficacy tended to participate in these NGO activities. In wartime, individuals tend to experience higher levels of psychological distress, being exposed to life threats, displacement, financial hardships, and witnessing atrocities [37]. Thus, to overcome the cumulative effect of exposure to dual stressors – war and cancer – individuals might apply coping behavior. Namely, they may attempt to manage internal or external stressors that they perceive as exceeding existing resources [38] by participating, for example, in NGO activities. Regarding T2, Benight and Bandura[12] argued that self-efficacy might determine coping behavior, given that self-efficacy beliefs regulate human functioning through cognitive, motivational, affective, and decisional processes. Thus, individuals who doubt their coping capabilities (low self-efficacy) might need to invest in new resources (i.e., NGO activities meant to alleviate wartime stress) to reduce the impact of the dual stressors that are associated with psychological distress, as COR theory suggests [9, 39]. We would recommend that future studies repeat this study model with a larger sample size to determine whether the findings can be replicated. Such replication would be particularly important given that only a small number of participants (*n* = 90) completed the questionnaires at both time-points in the present study.

The study’s second main finding was that participation in NGO activities meant to alleviate wartime stress at T2 was predicted by biological sex (female), lower self-efficacy, and participation in the aforementioned NGO activities at T1. Previous research has shown that compared to men, women are more likely to seek out sources of social support under stressful conditions, such as disease [40]. Moreover, stress responses among women may take the form of “tend-and-befriend” behavior, including joining groups to reduce susceptibility and developing social ties, especially with other women, to exchange resources and share responsibilities [41]. Thus, it might be that when confronted by dual challenges, such as cancer and war, women utilize NGO activities as an additional source of support, especially if the stressors trigger psychological distress and necessitate coping strategies. Further, as mentioned above, lower self-efficacy was found to be another predictor for engaging in NGO activities at both time-points. Wang and colleagues [42] demonstrated that enhancing social support systems positively affects self-efficacy; in other words, when individuals have lower self-efficacy, they might rely more on social support to compensate for their lack of confidence in managing their health condition or improving their mental health status. Indeed, according to COR theory [9], people invest resources for several reasons: to protect against resource loss, recover from losses, and gain resources. Finally, the third predictor for participation in NGO activities at T2 was participation in NGO activities at T1. An explanation for this finding might be found in previous research showing that social participation, including involvement in NGO activities, can enhance cognitive functioning and well-being; a positive feedback loop is thus created that motivates further participation [43]. The NGO’s wartime-adapted activities at T1 provided individuals with a supportive social network for addressing the dual stressors, including informational and emotional types of support. These activities fostered a sense of community which, in turn, motivated participants to continue engaging in the activities over time.

### Limitations

Although our findings provide valuable insights, certain limitations should be acknowledged. First, the study’s reliance on self-reported measures might have introduced bias due to participants’ subjective interpretations of questions or their current emotional state. Additionally, the study’s design does not allow for causal inferences; we cannot definitively conclude that the observed changes in psychological distress or engagement were directly caused by the NGO’s modified activities. Second, the size of the sample, which included participants who completed the questionnaires at both T1 and T2, may have impacted the study’s findings and may also limit the generalizability of the results. Furthermore, the study did not account for potential confounding variables such as participants’ previous experiences with trauma or access to other forms of wartime support; these variables could have influenced their psychological distress and engagement levels. Lastly, the focus on a single NGO may limit the applicability of the findings to other contexts or organizations.

### Implications

Participating in NGO activities tailored to cancer patients’/survivors’ needs may provide these individuals with a sense of normalcy, community, and support crucial for dealing with the added stress of war. In particular, individuals with lower confidence in their ability to manage their illness (i.e., lower self-efficacy) are more likely to seek such activities. Therefore, NGOs focusing on healthcare are encouraged to initiate activities adapted to wartime and illness needs, optimally ensuring the continued use of the organization’s services over time and engagement in the organization’s care activities during global crises. The higher participation of women in NGO activities (found in the current study) might indicate that men are less inclined to seek and engage in supportive community activities; as such, it is reasonable to suggest that more gender-specific activities are needed. The findings of Oppegaard and colleagues [44] also indicated that women diagnosed with cancer were more likely to seek emotional and communal coping mechanisms, whereas men reported a more individualistic approach.

From a broader perspective on NGOs and other support systems operating in similar crisis contexts, the current study has additional practical implications. First, it is recommended that NGOs develop flexible support frameworks that can rapidly adapt to changing circumstances, such as war or other crises. This adaptability would ensure the continuity of care and support, which is crucial for maintaining the psychological well-being of vulnerable populations, such as cancer patients. Additionally, it is essential to offer a range of support services that cater to different needs, including psychological support, physical health activities, and community engagement opportunities, to meet diverse needs and preferences. Second, NGOs can serve as a bridge between patients and formal healthcare systems, particularly when traditional healthcare services are disrupted during war or other crises [45]. This partnership can be vital in providing comprehensive care that addresses both the psychosocial and medical needs of patients.

## Conclusions

This study highlights the critical support provided by Halasartan to cancer patients/survivors during wartime, as it demonstrates how the activities provided were associated with a reduction in psychological distress over time. Moreover, the findings revealed gender differences in engagement in care activities during wartime, with women, as well as those with lower self-efficacy, more likely to participate. These insights emphasize the complex interplay between community support, individual coping mechanisms, and engagement over time in care activities. The study thus underscores the importance of tailored interventions to bolster the well-being of vulnerable populations during global crises.

## Data availability statement

No datasets were generated or analysed during the current study.

## Data Availability

No datasets were generated or analyzed during the current study.
